# Gene Editing Versus Morphants

**DOI:** 10.1089/zeb.2015.1114

**Published:** 2015-10-01

**Authors:** Paul A. Morcos, Alexandra C. Vincent, Jon D. Moulton

**Affiliations:** ^1^ OligoExpert, Corvallis, Oregon.; ^2^ Gene Tools, LLC, Philomath, Oregon.

## Dear Editor:

Kok *et al*. conclude that results with Morpholino oligos, which are not recapitulated by natural mutations or gene editing mutations, must be due to off-target effects and, when morphants and mutants differ, they contend that antisense oligo studies should be disregarded.^1^ Morpholinos and gene editing mutagenesis are very different technologies and, even if both work perfectly, may yield different results targeting the same gene. Rossi *et al.* showed in zebrafish with a gene either knocked out or knocked down that some untargeted genes altered their expression in the mutant but not the morphant.^2^ The morphants showed a strong phenotype but the mutants, with their compensatory expression changes, did not show the phenotype. Another knockdown technique, CRISPRi, phenocopied the Morpholino result. They further showed that when Morpholinos were used in zebrafish heterozygous for the mutation, the morphant phenotype was partially suppressed, which was not expected if the morphant phenotype were primarily due to off-target RNA interaction. Gene editing technologies cause permanent changes in DNA. Morpholinos work on RNA, can attenuate targets that may be lethal when knocked out with gene editing, and cause transient and concentration-dependent knockdowns. We argue that different reported outcomes of Morpholinos and gene editing may be due to the differences in timing and site of action in addition to overdosing, lack of p53 controls, and lack of specificity controls when using Morpholinos.

Antisense oligos can give rise to off-target effects, especially at high concentration. However, screening Morpholino target sequence with BLAST and using proper dosing routinely yields valuable and reproducible results. To showcase their hypothesis, Kok *et al.* describe a phenotype from a Morpholino used in a *megamind* null mutant.^1^ The MO dose was 20ng/embryo, higher than the 5ng dose Ulitsky *et al.* reported for their *megamind* morphant.^3^ Further, Lin *et al.* used both splice-targeted and conserved-region-targeted Morpholinos against *megamind* at 1ng and found no morphological phenotype.^4^ Notably, 1ng caused a behavioral phenotype and made the wild-spliced RT-PCR gel band disappear; while in the *megamind* mutant this phenotype has not been reported, behavior might not have been assessed. Lin *et al.* show a *megamind*-targeted Morpholino alters RNA processing and yields phenotypic data when used at a dose below that causing hydrocephaly; Kok *et al.* did not report testing a series of lower doses.

Kok *et al.* continue presenting the merits of gene editing technologies by comparing selected experimental results for specific genes: (a) whose RNA transcripts are blocked by Morpholinos, versus (b) whose DNA sequences are partially or fully deleted by gene editing technologies.^1^ They conclude that the vast majority of published Morpholino results are due to off-target effects because the Morpholino and gene editing results do not match, however Rossi *et al.* show compensation might cause the difference.^2^ In addition, most of the Morpholino work chosen for comparison did not report controlling for specificity by phenocopying using non-overlapping Morpholinos targeted to the same RNA and did not report screening for activation of the p53-mediated apoptosis cascade. Oligo specificity is commonly confirmed by phenocopying with non-overlapping Morpholinos or using rescue mRNAs. Morpholinos confirmed with these techniques and used at optimized dosage are very specific for their targeted RNA sequences.

Kok *et al.* advocate rejecting Morpholino results unless a knockdown phenotype is confirmed against a mutant.^1^ This is causing some reviewers of papers and grants to discount Morpholino experiments and insist DNA manipulations be used instead of or in addition to Morpholinos. We believe this is counter-productive for the developmental biology community. Generating morphants with Morpholinos is a well-validated technology with solid targeting rules and multiple strategies available for rigorously confirming results.
